# Psychosocial impact of climatotherapy in young patients with psoriasis: a 3-month cohort study

**DOI:** 10.3389/fmed.2024.1458394

**Published:** 2024-10-29

**Authors:** Max Nordgren, Albert Duvetorp

**Affiliations:** ^1^Diagnostic Center Dermatology, Malmö, Sweden; ^2^Deparment of Dermatology, Skåne University Hospital, Malmö, Sweden; ^3^Leo Foundation Skin Immunology Research Center, Copenhagen University, Copenhagen, Denmark; ^4^Department of Clinical Sciences, Lund University, Malmö, Sweden

**Keywords:** climatotherapy, heliotherapy, phototherapy, psoriasis, psychosocial factors, prospective study

## Abstract

Ultraviolet rays are known to have positive effect on psoriasis, but the cold climate and latitude in Northern Europe reduce access to the sun. Climatotherapy is a treatment modality where the patient is relocated to a warmer region with a high ultraviolet (UV) index. Young patients with psoriasis can be particularly burdened by the disease, and studies have shown an association between psoriasis and mood disorders. Patients who have undergone climatotherapy often report beneficial psychological effects after treatment, but this has not yet been studied. To explore the psychosocial impact of climatotherapy, an observational study was designed. Thirty-four participants (median age of 24 years) underwent 3 weeks of treatment in Gran Canaria (Spain) and responded to questionnaires assessing the psychosocial aspects of the disease. Climatotherapy was associated with significantly reduced scores of HADS, PSS-10, PSQ, EQ-VAS, DLQI, and itch intensity at the end of and at 3 months after the end of treatment compared to before treatment onset. The results suggest that climatotherapy not only exerts effects on psoriasis skin disease but also improves symptoms of anxiety, depression, perceived stigmatization, stress, quality of life, self-assessed overall health perception, and itch intensity. The results can be of use in the decision-making process when choosing a suitable treatment for young patients with psoriasis.

## 1 Introduction

Ultraviolet rays (UV rays) are known to have a positive effect on psoriasis, but the cold climate and the lower UV indexes of northern and southern latitudes of the Earth reduce the possibility of inhabitants with psoriasis in these regions taking advantage of the sun. Studies indicate that the prevalence of psoriasis in Northern Europe is considerably higher than the prevalence seen in regions closer to the Equator (1, 2). Patients with psoriasis have an increased risk of depression and suicidal thoughts and have reported a reduction in mental and physical functioning comparable to that seen in heart disease, arthritis, diabetes, and cancer (3–5). Individuals with early onset of psoriasis (age < 20 years) are significantly more depressed and anxious than patients with the late-onset disease (6). Climatotherapy (CT) is a treatment modality where the patient is temporarily relocated to a warm region with a higher UV index and exposed to the sun according to skin type with an incremental dosage regimen. Several studies on CT have proven this effect of treatment modality on psoriasis (7–11). Exposure to UV rays decreases inflammation in psoriasis by various mechanisms, inducing T-cell apoptosis, increasing the proportion of regulatory T cells in skin and blood, and reduction in mediators of the Th17 pathway and chemotaxis (12, 13).

A few studies have also investigated the impact on DLQI, EQ-5D, and EQ-VAS. In a Danish study with 18 participants (mean age of 52.2 years) attending CT at the Dead Sea, mean DLQI was 13.9 before CT and reduced to 2.0 at the end of treatment, EQ-5D increased from 0.79 to 0.90, but there was no difference in EQ-VAS (8). A follow-up assessment was performed when the first visible sign of psoriasis reappeared (mean time 94 days). Neither DLQI, EQ-5D, nor EQ-VAS was reduced from baseline at this assessment. A study with 49 participants (mean age of 51.9 years) investigating medium- to long-term effects of CT found that DLQI was 16.1 before CT and reduced to 10.6 at a follow-up time of 3 to 6 months after treatment (9).

CT is a treatment modality that is currently being replaced by modern systemics; hence, it could be important to map effects beyond the skin and DLQI. Patients who have undergone CT often report beneficial psychological effects after treatment, but this observation has not been thoroughly studied. In this observational study, we investigated whether CT impacts psychological symptoms in young patients with psoriasis using a questionnaire-based approach.

## 2 Materials and methods

### 2.1 Subject inclusion and climatotherapy intervention

The patient organization *Ung med psoriasis* has organized annual CT trips to Gran Canaria since 1987. Applications are open for all individuals with psoriasis in Sweden between the ages 15 and 35 years. Applications include an evaluation by the applicant’s dermatologist, which enables for the selection of participants with higher needs, mainly based on PASI and DLQI scores (see Supplementary Figure S1 for the application form). Applicants with younger ages (age group 15–25 years) are also prioritized. CT used to be financed through the public healthcare system but with the introduction of more efficient systemic drugs, this is no longer so in many regions of Sweden. Although subsidized through private grants and grants to the patient organization, the CT of *Ung med psoriasis* trips is in part funded by participant fees (approximate cost of 515 euros per participant).

Patients of three 3-week-long CT trips to Gran Canaria during 2018, 2019, and 2022 (in June and July) were included in a prospective observational cohort study. No CT trips were arranged in 2020 and 2021 due to the COVID-19 pandemic. All participants were living in Sweden. Inclusion criteria were being a participant in the *Ung med psoriasis* CT trip, being able to understand study information, and giving informed consent. The exclusion criterion was having participated in the study a previous year. The intervention program included daily scheduled sunbathing with durations depending on skin type (incremented every third day until day 8 if sun exposure has been tolerated well), physical activity on weekdays, educational lectures, social events, motivational discussions, and assisted topical exfoliative treatment. The approximate total sunbathing time over the 3 weeks would be 805 + 805 min for Fitzpatrick skin type II, 1,610 + 1,610 min for skin type III, and 2,190 + 2,190 min for skin type IV (anterior and posterior sides of the body). Participants were encouraged to always use sunscreen minimum SPF 50 with UVA and UVB filter on non-lesional skin and lesional skin after the daily scheduled sunbathing had been completed. Participants responded to questionnaires Hospital Anxiety and Depression Scale (HADS) (14), Perceived Stress Scale 10 (PSS-10) (15), Perceived Stigmatization Questionnaire (PSQ) (16), Dermatology Life Quality Index (DLQI) (17), EQ visual analog scale (EQ VAS) (*The EuroQol Group*), and itch intensity graded from 0 to 10 at three time points.

**Figure 1:**
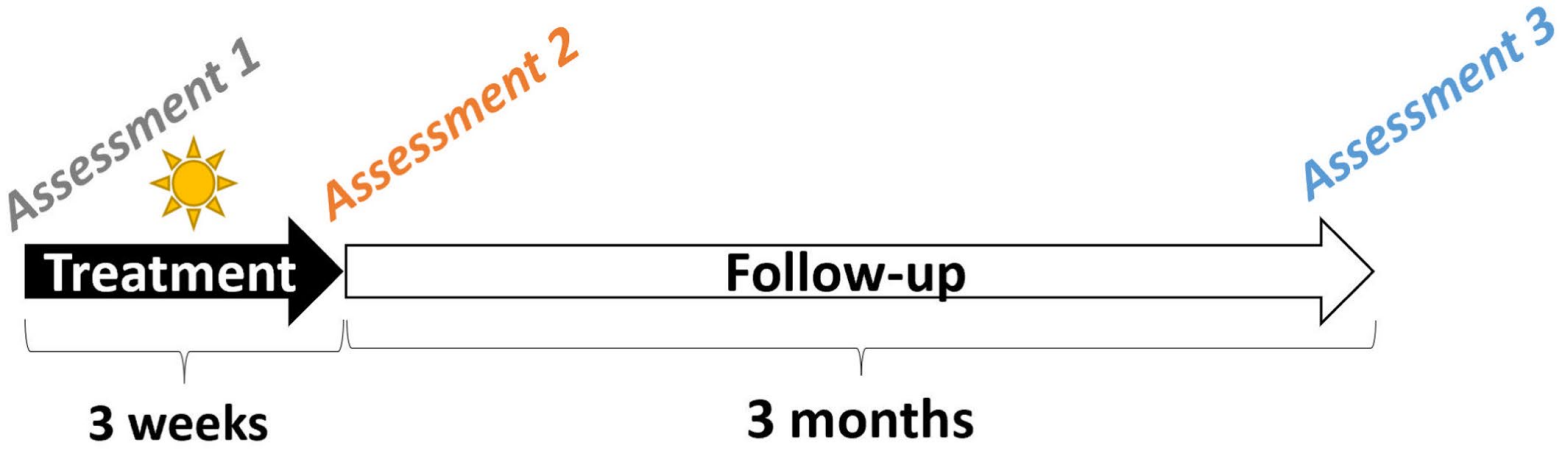
Assessments. Timeline illustrating assessment 1 (before the start of treatment), assessment 2 (end of treatment), and assessment 3 (3 months after the end of treatment).

The time points of assessment were before start of treatment (on the day of arrival), end of treatment (on the day or day before departure), and 3 months after the end of treatment (Figure 1). Psoriasis Assessment Severity Index (PASI) was evaluated before treatment and at end of treatment by AD (consultant dermatologist). HADS is a questionnaire frequently used in healthcare to assess symptoms of anxiety and depression during the previous week. PSS-10 is a questionnaire developed to assess stress levels in young people above 12 years of age and adults the previous month. PSQ is a questionnaire originally developed to assess stigmatization behaviors among burn survivors but is also used for the assessment of stigma related to other visible skin diseases. The study was registered in the clinical trial registry *Deutsche Register fur Klinische Studien* with DRKS-ID DRKS00014916 as at the time being no equivalent register was available in Sweden.

### 2.2 Statistical analysis and handling of missing data

Pre-treatment scores were compared with scores at the end of treatment and 3 months after the end of treatment. Significance levels were calculated with the Friedman test and *post-hoc* two-tailed Wilcoxon signed-rank test using IBM SPSS Statistics v. 27. A *p*-value of <0.05 was considered significant. Descriptive statistics were performed to assess medians and interquartile ranges.

Two participants failed to answer all the questions in one of the questionnaires at one time point (HADS after treatment for one participant and PSQ after treatment for the other participant). These two participants were excluded from the analyses of HADS and PSQ, respectively. Several participants failed to answer one isolated question in a questionnaire. This was handled by giving a score in the direction of disadvantage the significance level to avoid false significance.

### 2.3 Ethics

The study was approved by the Ethics Review Board in Lund, Sweden (Dnr 2018/378).

## 3 Results

Thirty-four of 35 invited research subjects (participants of CT) accepted participation giving their written consent and were included in the study. One participant did not complete the 3-month follow-up and was classified as lost to follow-up (and excluded from analysis). Demographics and clinical characteristics of the cohort at baseline can be seen in Table 1.

**Table 1:** Patient demographics and clinical characteristics at baseline.

Number of participants	34
Males, n (%)	13 (38)
Females, n (%)	21 (62)
Age, median (range)	24 (15–34)
Duration of psoriasis, years, median (range)	10.4 (0–22)
BMI, kg/m^2^, median (range)	29 (16.5–52.8)
Previous climatotherapy, n (%)	20 (59)
Was of 1 trip, n (%)	9 (26)
Was of 2–3 trips, n (%)	5 (15)
Was of >3 trips, n (%)	6 (18)
Heredity of psoriasis 1st degree, n (%)	11 (32)
Heredity of psoriasis 1st to 2nd degree, n (%)	19 (56)
Heredity of psoriasis 1st to 4th degree, n (%)	22 (65)
Use of systemic treatment, n (%)	15 (44)

BMI, body mass index.

**Figure 2:**
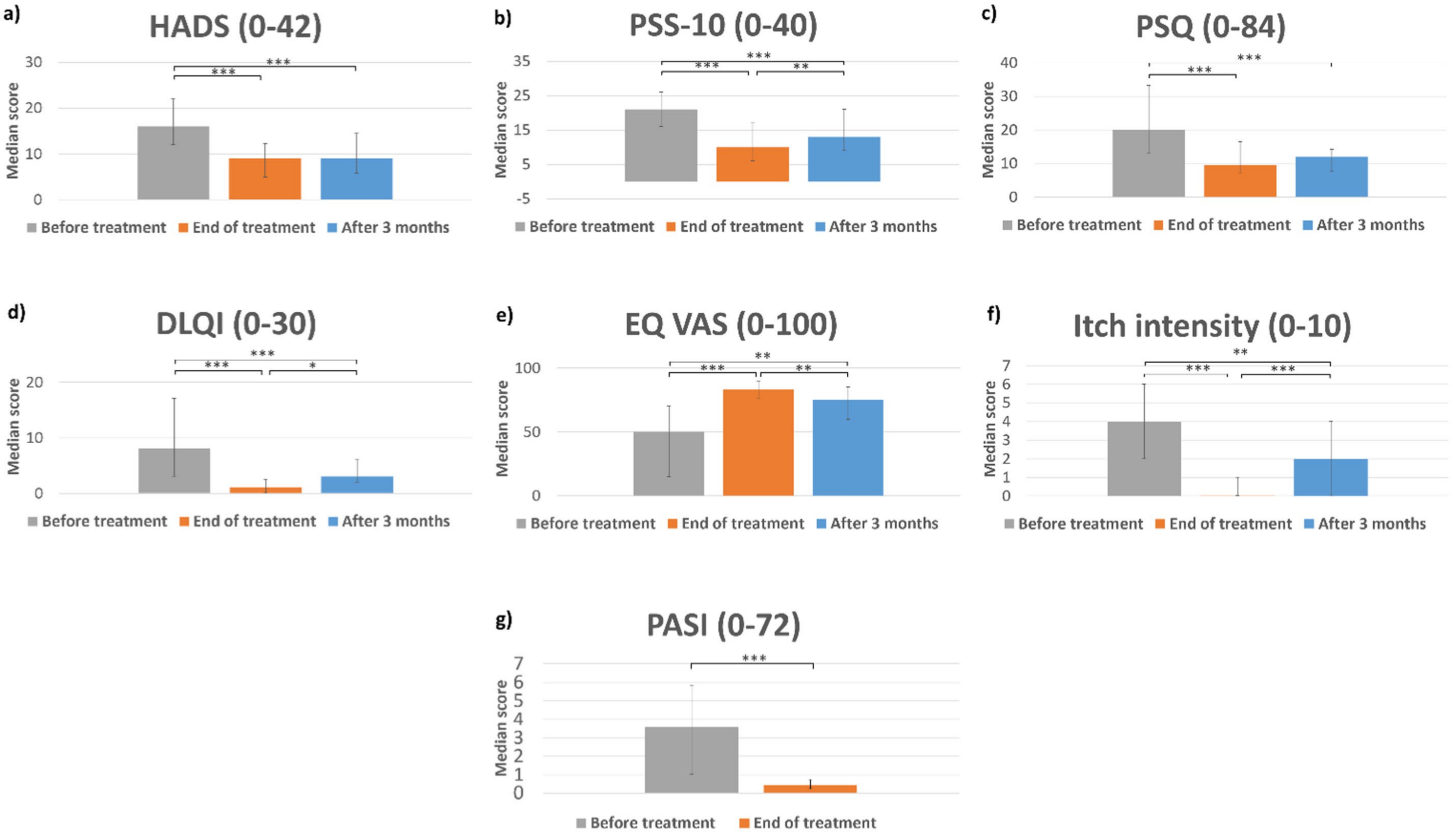
Results. Bar charts showing median scores before the start of treatment, at the end of treatment, and at 3 months after the end of treatment for (a) Hospital Anxiety and Depression Scale (HADS), (b) Perceived Stress Scale 10 (PSS-10), (c) Perceived Stigmatization Questionnaire (PSQ), (d) Dermatology Life Quality Index (DLQI), (e) EQ visual analog scale (EQ-VAS), (f) itch intensity, and (g) Psoriasis Assessment Severity Index (PASI). Error bars represent the first and third interquartiles. Values in parentheses in chart titles represent theoretical minimum and maximum scores for scales. *p < 0.05, **p < 0.01, and ***p < 0.001.

CT was associated with a significantly reduced score of HADS, PSS-10, PSQ, DLQI, and itch intensity at the end of treatment and at 3 months after the end of treatment compared with before the start of treatment. EQ VAS was significantly increased at the end of treatment and at 3 months after the end of treatment. These significant changes correspond to decreased symptoms of anxiety, depression, stress level, stigmatization, and itch level and increased dermatologic-orientated life quality and overall health quality. PASI was assessed before and at the end of treatment and was significantly reduced. See Figure 2 for scores, interquartile range, and significance levels. For more precise details, see Supplementary Table S1.

## 4 Discussion

To our knowledge, no previous studies have investigated the psychological impact of CT with instruments specifically validated for anxiety, depression, stress, and stigmatization. In this study, we show that CT results in a positive psychosocial impact with sustained effects for at least 3 months post-treatment follow-up.

In the current study, the effects on DLQI and EQ-VAS were more pronounced and sustained also at assessment 3 months after treatment compared to previous studies (8, 9). Possible reasons for discrepancies between the current study and previous publications might be differences in the age of participants (younger in the current study), differences in disease severity (lower PASI in the current study), and differences in the intervention programs. CT trips performed by *Ung med psoriasis* are directed toward younger individuals, with a focus on getting to know peers with the same disease, education, exercise, self-empowerment, and discussing disease impact in small groups apart from clearing the skin from psoriasis. *Ung med psoriasis* CT group leaders often have psoriasis themselves and can as such figure as older role models for younger participants.

Normative data on HADS for randomly chosen adolescents and young adults from the general population in Sweden aged 13 to 23 years were published in 2006 (18). HADS median scores were sorted out separately for anxiety and depression with scores of 5.0 and 2.0, respectively, for the mail responders and 4.0 and 2.0, respectively, for telephone responders. This can be compared to our study with median scores of 10.50 for anxiety and 5.5 for depression before the start of treatment and 6.5 for anxiety and 2.5 for depression 3 months after treatment. This indicates that our psoriasis cohort suffered more from anxiety and depressive symptoms than the general population before the start of treatment but had scores similar to the general population 3 months after treatment. Normative data for PSS-10 are also available for the Swedish population published in 2013 (19). The age group closest to our cohort was aged 18 to 34 years who scored a mean of 15.6 (the median was not present in the study). The mean for our cohort was 22.0 (median 21.0) before the start of treatment, 11.5 (median 10) after the end of treatment, and 15.5 (median 13.0) 3 months after treatment. This indicates that our cohort suffered from more stress than the general population before the start of treatment but achieved a stress level comparable to the general population after treatment and at the 3 months post-treatment follow-up.

Psychosocial parameters have been evaluated in psoriasis studies investigating many other treatment modalities, and it is appealing to compare our results with such studies. Direct comparison is difficult as methods and study populations often are divergent. An observational study on narrowband-UVB (nb-UVB) phototherapy evaluated the psychosocial parameters DLQI and itch intensity (also included in our study) on participants with psoriasis including 3 months after the end-of-treatment follow-up (20). The baseline was in part comparable to our study with a mean DLQI of 10.1 (*vs* 10.2) and mean itch intensity of 4.4 (*vs* 4.1). At 3-month follow-up, mean DLQI was 6.3 and itch intensity 3 in comparison with DLQI in our study being 4.1 (median 3.0) and mean itch intensity 2.6 (median 2.0). CT may add more to the quality of life than nb-UVB, but differences are small, and a head-to-head trial would be needed to confirm this. Some discrepancies in the nb-UVB study compared to our study are age (mean 51 vs. 23.5), baseline PASI (self-administered PASI 11.7 vs. mean 4.5), and higher use of systemic treatments in our study. Many phase 3 trials for biologic therapies including TNF inhibitors, IL23 inhibitors, and IL17 inhibitors have included secondary outcomes including symptoms of depression and anxiety apart from quality of life. As such guselkumab (IL23i) and adalimumab (TNFi) have been shown to have effects on DLQI and HADS scores even after adjusting for indirect effects mediated by skin improvement (21). The mean HADS-A and HADS-D improvement at week 16 in the Phase 3 VOYAGE 2 study (22) was −1.7 and − 1.6 for guselkumab and − 1.1 and − 1.2 for adalimumab—which are somewhat modest improvements compared to the results of our study. A randomized trial evaluated the benefit of a 3-week course of thermal spa therapy (balneotherapy) on psychosocial parameters and compared it with a group having usual treatment (23). All participants were adults with plaque psoriasis and had a DLQI of >10 at inclusion (mean 16.7). The assessment was done 4.5 months after the intervention. In the spa group, 66.1% achieved DLQI ≤10 compared with 41.4% in the placebo group. In addition, itch intensity (pruritus VAS) and PSS were assessed. There was no significant difference in PSS, but itch intensity significantly decreased from 6.7 to 4.3 in the treatment group compared with 6.8 to 5.4 in the control group. The higher impact on psychosocial parameters in our study could be attributed to a shorter follow-up time and younger age of our participants, but it is also possible that the effect of CT is superior to that seen with balneotherapy.

PASI was reduced to a median of 0.4 and itch intensity to a median of 0.0 after CT (Figure 2). PASI was not assessed at 3 months as participants were recruited from all of Sweden and assessment was not regarded as logistically feasible. The increment in itch seen at 3 months could suggest that some participants may have had a partial relapse of skin disease. Medium-to long-term PASI results after CT have been studied before which has shown that one-fifth of patients will have sustained treatment effect at 4- to 6-month follow-up (PASI75) (9). The median PASI score before the start of treatment was lower in the current study than previous studies on CT. This is probably attributed to the high prevalence of systemic treatments in this cohort (44%). The higher prevalence of systemic treatment is probably attributed to the general change in how psoriasis is treated in Sweden (national treatment goals are currently PASI <3 and DLQI ≤5). CT participants were encouraged to continue their baseline psoriasis treatment during the follow-up period. The psychosocial impact seen despite a relatively low PASI score before the start of treatment may suggest that not only scheduled sunbathing but also the other interventions of CT contribute to positive psychological effects. Future studies with a mixed-method approach including both quantitative and qualitative assessments may clarify which components of CT that exert the effects found. It is possible that such studies could facilitate the design of interventions to help young patients with the psychosocial burden of disease also without travel.

## 5 Limitations

As in most studies on CT, the participants served as their own controls, which arguably is a weakness. In a randomized trial with a separate control group, an “unhappiness-bias” might emerge. A degree of a negative psychosocial impact could be attributed to the disappointment of being assigned to the control group and not being able to join the CT. Participants of *Ung med psoriasis* CT trips apply to the trips themselves (although a dermatologist assessment is obligatory). It is assumable that individuals only will apply if they believe that the treatment modality could be of benefit to them, which could lead to a selection bias. Furthermore, the lack of a 3-month PASI assessment makes it difficult to compare psychosocial effects with effects on skin disease activity at this time point, which is a weakness.

## 6 Conclusion

The results suggest that CT could be an attractive treatment for young individuals with psoriasis with a marked psychosocial disease burden. Further studies are needed to determine what mediates the observed effects and to determine whether the effects are still standing beyond 3-month follow-up.

## Data Availability

The original contributions presented in the study are included in the article/supplementary material, further inquiries can be directed to the corresponding author.

## Supplementary material

SUPPLEMENTARY FIGURE S1 Application form.
