# Supplementary figures and images for: Psychosocial impact of climatotherapy in young patients with psoriasis: a 3-month cohort study
Source: Front Med (Lausanne). 2024 Oct 29;11:1458394. doi: 10.3389/fmed.2024.1458394 (PMC11554493; doi:10.3389/fmed.2024.1458394)

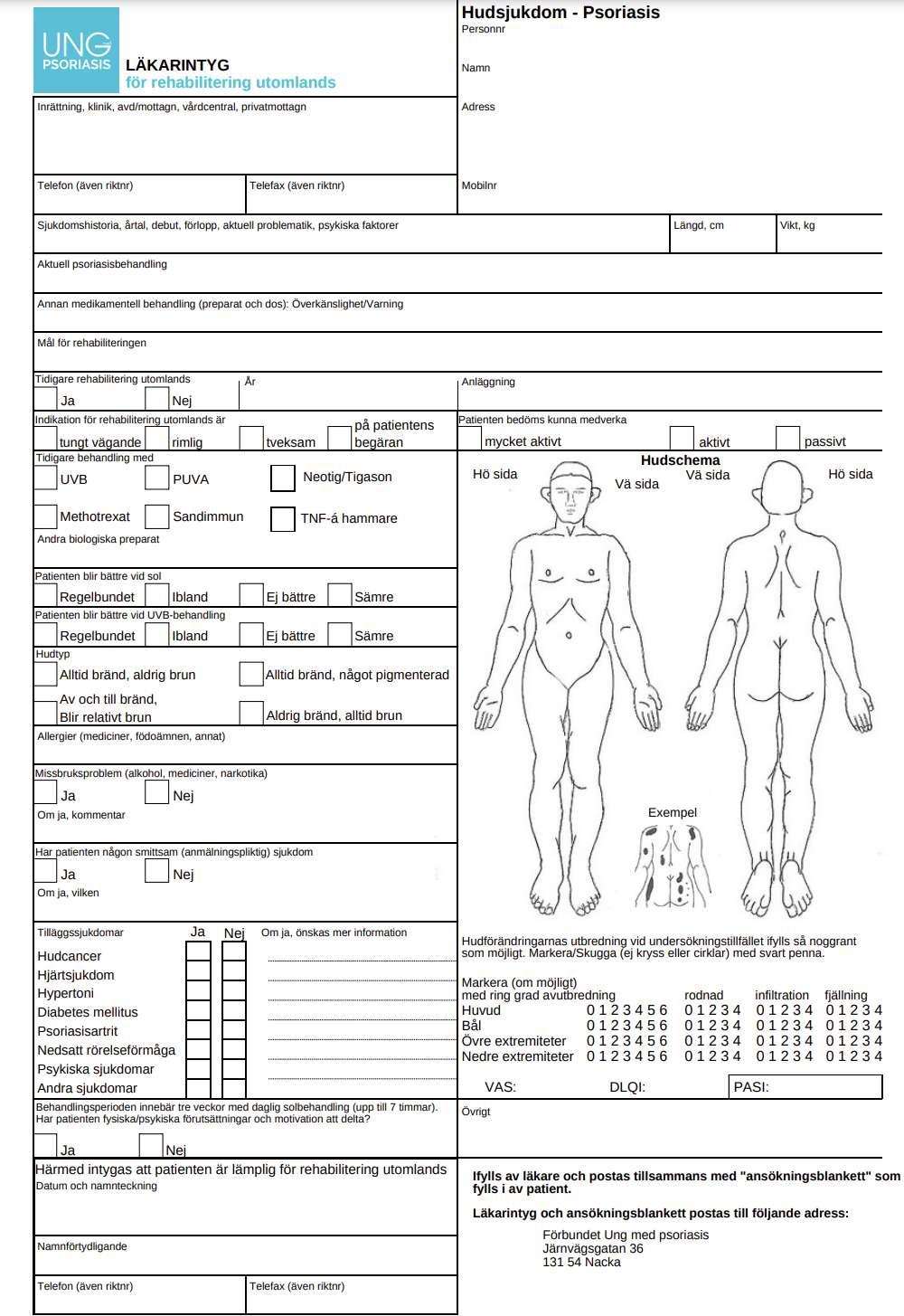

Supplement: SUPPLEMENTARY FIGURE S1 — Application form. [file Image_1.TIF]
