# Supplementary material for: Psychosocial impact of climatotherapy in young patients with psoriasis: a 3-month cohort study
Source: Front Med (Lausanne). 2024 Oct 29;11:1458394. doi: 10.3389/fmed.2024.1458394 (PMC11554493; doi:10.3389/fmed.2024.1458394)
Supplement: Supplementary file 2 [file Table_1.pdf]

**Table SI. Table with detailed results**

| | Medians | | | | Post-hoc | | | | | | | | | |
|---|---|---|---|---|---|---|---|---|---|---|---|---|---|---|
| | B treatment | E treatment | 3 m after E | P Friedman test | P-value B vs. E | P-value B vs. 3 m | P-value E vs. 3 m | B Q1 | E Q1 | 3 m Q1 | B Q3 | E Q3 | 3 m Q3 | Response rate |
| PASI (0-72) | 3.6 | 0.4 | N/A | N/A | 0.00000053 | N/A | N/A | 1.0 | 0.2 | N/A | 5.8 | 0.7 | N/A | 100% |
| Nr. of skin locations (0-7) | 4.0 | 1.0 | 3.0 | 0.00000078 | 0.000012 | 0.00041 | 0.0020 | 3.0 | 1.0 | 2.0 | 5.0 | 2.0 | 4.0 | 97% |
| Itch intensity (0-10) | 4.0 | 0.0 | 2.0 | 0.000000020 | 0.0000080 | 0.0055 | 0.00033 | 2.0 | 0.0 | 0.0 | 6.0 | 1.0 | 4.0 | 97% |
| DLQI (0-30) | 8.0 | 1.0 | 3.0 | 0.0000000022 | 0.00000099 | 0.000016 | 0.036 | 3.0 | 0.0 | 2.0 | 17.0 | 2.5 | 6.0 | 97% |
| EQ VAS (0-100) | 50.0 | 83.0 | 75.0 | 0.000000012 | 0.0000010 | 0.00058 | 0.0079 | 35.0 | 76.0 | 60.0 | 70.0 | 90.0 | 85.0 | 97% |
| PSS-10 (0-40) | 21.0 | 10.0 | 13.0 | 0.000000011 | 0.0000020 | 0.00015 | 0.0020 | 16.0 | 6.0 | 9.0 | 26.0 | 17.0 | 21.0 | 97% |
| PSQ total (0-84) | 20.0 | 9.5 | 12.0 | 0.0000000014 | 0.0000050 | 0.0000020 | 0.46 | 13.0 | 7.0 | 7.8 | 33.3 | 12.3 | 14.3 | 94% |
| HADS Depression (0-21) | 5.5 | 3.0 | 2.5 | 0.00022 | 0.00057 | 0.0028 | 0.91 | 2.0 | 2.0 | 1.0 | 8.0 | 5.0 | 5.3 | 94% |
| HADS Anxiety (0-21) | 10.5 | 6.0 | 6.5 | 0.00000030 | 0.0000040 | 0.000061 | 0.065 | 8.0 | 2.0 | 4.0 | 14.5 | 8 | 9.3 | 94% |
| HADS Total (0-42) | 16.0 | 9.0 | 9.0 | 0.0000010 | 0.0000040 | 0.000071 | 0.40 | 12.0 | 5.0 | 5.7 | 22.0 | 12.3 | 14.5 | 94% |
| BMI, (kg/m$^2$) | 29.4 | N/A | 28.4 | N/A | N/A | 0.83 | N/A | 23.7 | N/A | 24.1 | 31.2 | N/A | 31.3 | 91% |

B: Before; E: End of; 3 m: 3 months; Q: First interquartile; Q3: Third interquartile
