# Supplementary material for: Psychosocial impact of climatotherapy in young patients with psoriasis: a 3-month cohort study
Source: Front Med (Lausanne). 2024 Oct 29;11:1458394. doi: 10.3389/fmed.2024.1458394 (PMC11554493; doi:10.3389/fmed.2024.1458394)
Supplement: Supplementary file 3 [file Table_2.pdf]

Table SII. Table with individual data

**Part.**	**Systemic**	**PASI S**	**PASI E**	**HADS S**	**HADS E**	**HADS 3m**	**PSS-10 S**	**PSS-10 E**	**PSS-10 3m**	**PSQ-10 S**	**PSQ-10 E**	**PSQ-10 3m**	**DLQI S**	**DLQI E**	**DLQI 3m**	**EQ VAS S**	**EQ VAS E**	**EQ VAS 3m**	**Itch S**	**Itch E**	**Itch 3m**
1	No	4.6	0.6	16	6	4	17	6	4	18	10	3	17	3	4	75	85	75	5	0	2
2	Etanercept	3	0	ex	md	ex	40	8	6	28	16	12	20	1	2	30	85	100	6	0	2
3	No	2.4	0.4	14	4	5	26	7	11	19	3	13	13	3	3	40	90	80	6	0	2
4	No	4.6	0	27	23	18	25	24	33	34	37	34	21	1	19	18	100	35	4	3	1
5	No	2.6	1.1	17	12	10	23	14	11	46.5	9	28	19	2	4	30	78	85	7	0	1
6	No	3.2	0.2	7	9	7	16.5	14	13	ex	md	ex	3	0	3	70	90	80	3	2	1
7	No	2.4	0.2	5	5	1	14	5	5	12	1	0	3	0	1	70	90	87	2	0	2
8	No	6	0.6	20	3	4	26	10	13	45	10	19	21	2	3	35	76	86	8	0	2
9	Methotrexate p.o.	2	0	27	20	35	31	20	34	34	12	15	9	1	3	50	75	70	0	0	2
10	No	3.2	0.2	5	0	3	10	2	4	11	8	7	6	0	2	90	90	90	1	0	2
11	No	9.2	0.5	5	3	4	10	3	7	2	0	0	1	0	0	60	80	100	0	0	2
12	Methorexate s.c.	1.4	0	24	11	27	36	8	33	14	6	12	2	3	7	80	100	40	3	0	1
13	No	7.2	0.7	9	2	10	16	10	20	22	8	14	8	2.5	3	50	83	59	2	0	2
14	No	4.8	0.4	14	9	12	19	10	11	31	21	22	5	1	5	50	100	40	4	0	1
15	No	9.2	1	30	26	23	33	27	32	33	7	13	23	4	6	10	70	60	4	2	1
16	Adalimumab	3.9	1.9	12	9	6	16	18	13	13	11	9	1	0	1	70	84	80	3	3	1
17	No	8.3	1.2	21	12	12	25	22	23	33	24	29	20	3	14	43	85	25	8.5	0	1
18	No	1	0	18	5	6	14	5	8	7	0	0	3	0	3	60	95	70	6	0	1
19	Ustekinumab	4.4	0.4	ex	ex	md	ex	ex	md	ex	ex	md	ex	ex	md	ex	ex	md	md	ex	md
20	No	3	0.7	26	14	24	36	11	25	51.5	5	26	20	0.5	7	30	72.5	90	2	0	1
21	No	4.7	0.3	16	7	6	24	14	21	8	12	8	7	1	2	50	80	70	3	0	1
22	Brodalumab	6	1	24	18	17	28	17	24	13	12	9	10	3	6	55	65	40	5	3	1
23	Secukinumab	0.4	0	6	11	7	0	0	0	5	4	4	3	0	1	86	94	100	0	0	2
24	Ustekinumab	0	0	12	10	16	16	15	25	9	9	7	1	0	0	92	96	79	0	0	2
25	No	22	0.9	30	7	12	37	6	13	56	8	11	26	1	2	0	89	89	10	0	1
26	Secukinumab	1	0.2	17	7	6	20	10	14	37	32	20	9	2	6	50	76	50	5	0	1
27	Secukinumab	9.7	0.2	16	13	16	21	10	20	31	9	12	13	2	3	50	80	80	10	3	1
28	Ustekinumab	1.1	0	13	7	5	20	17	8	17	9	8	7	1	1	70	70	71	0	0	2
29	Ustekinumab	3.2	0.2	13	9	7	15	11	9	20	17	14	3	1	1	70	80	80	0	0	2
30	Secukinumab+ Methotrexate p.o.	0.9	0.2	22	19	13	27	18	17	14	13	7	9	9	7	30	71	60	8	6	1
31	Guselkumab	1.9	0.4	14	2	8	21	4	13	35	11	10	7	0	2	60	80	70	5	0	2
32	No	4.2	1.3	19	13	3	25	14	12	20	14	14	8	4	3	35	60	70	6	3	1
33	No	5.1	0.7	12	7	10	17	17	17	23	12	12	4	1	4	70	80	81	3	1	1
34	Etanercept	6	0.4	22	0	14	21	2	13	13	7	9	16	0	7	25	91	59	5	0	1

Part.: Particioant, Systemic: Systemic treatment (there was no difference at individual basis between use of systemic treatment at start and at 3 months), B: Before; E: End of; 3m: 3 months, p.o.: Per oral (oral intake), s.c.: Subcutaneous, md: Missing data, Ex: Excluded due to missing data in same type of questionnaire
